# Design and Research of 2-DOF Piezoelectric Nanopositioning Stage with Multiple Displacement Amplifiers and Decoupling Beams

**DOI:** 10.3390/mi17040484

**Published:** 2026-04-16

**Authors:** Jing Yin, Chen Zhou, Xiaoting Chen, Dongcai Liu

**Affiliations:** Anhui Province Key Laboratory of Simulation Calculation and Design for Electronic Information System, Hefei Normal University, Hefei 230601, China; zhouchenst@163.com (C.Z.); c_t_chen@163.com (X.C.); liudc_hf@foxmail.com (D.L.)

**Keywords:** piezoelectric nanopositioning stage, compliant mechanism, displacement amplifier, decoupling mechanism, lever amplifier

## Abstract

A piezoelectric (PZT) nanopositioning stage with large stroke and low crosstalk is relatively appealing for microdisplacement operation. Rhombus and lever amplifiers are used to increase the overall displacement amplification ratio (DAR), and the symmetry of the amplification structure reduces the coupling error. At the same time, decoupling beams are used to balance the stiffness in the x- and y-directions, thereby reducing the crosstalk of the stage. Theoretical analysis, kinematics modeling, and finite element simulation were carried out to verify the feasibility of the PZT nanopositioning stage, and an experimental prototype was manufactured. The prototype test results indicate that the workspace of the stage is 312 μm × 312 μm, the DAR is approximately 9.3, and the first natural frequency is approximately 76 Hz. Moreover, the area efficiency is 6.03, which indicates that the stage is compact. The results prove that the developed stage possesses a good property for microdisplacement operation.

## 1. Introduction

Compliant mechanisms based on flexible beams are widely used in various disciplines and industrial applications [1], including nanopositioning [2,3], micrograsping operation [4], and fast servo tools for precision manufacturing [5]. In these applications, large motion ranges, fast responses, and high-precision nanopositioning platforms are urgently required. In order to achieve a larger workspace, preliminary research has been conducted as follows.

Some common solutions include selecting a large-stroke actuator. Typically, due to its advantage of large stroke, a voice coil motor (VCM) is often used as the actuator [6]. Shang et al. presented a two-degree flexure-based micropositioning stage with a workspace of 1.8 mm × 1.78 mm. However, the VCM-actuated positioning stage could not realize positioning in nanoscale due to the low resolution of the VCM actuator [7].

Piezoelectric (PZT) actuators, which convert electrical energy into mechanical energy, are often used as actuators in compliant mechanisms due to their fast frequency response, nanoscale resolution, and compact structure, considering resolution. However, the strain of PZT actuators is typically only 0.1%. An appropriate displacement amplifier must be designed to increase the stroke of the PZT-actuated platform. Yong et al. proposed a compliant two-degrees-of-freedom (2-DOF) nanopositioning stage with lever displacement amplification, and the displacement amplification ratio (DAR) of the two axes was approximately 2.5, and the workspace was 10 μm × 10 μm with a high resolution of 39 nm [8]. Wang et al. designed a PZT-actuated compliant microgripper with multiple-lever amplifiers, and the DAR was 22.3 [9]. Tang et al. developed a piezo-actuated flexible 2-DOF micromanipulator with a millimeter workspace of 3.1273 mm × 26.5°, and the DAR was 31 [10]. Zhu et al. developed a piezo-actuated parallel mechanism with a bridge-type mechanism and two-stage leverage mechanisms for increasing the DAR as high as 35.68 [11]. However, with the increase in lever series and DAR, the dynamic performance of the flexible mechanism becomes worse for the recognized tradeoff between dynamic frequency and motion range in the design of flexible mechanisms, thereby greatly limiting their use for simultaneous large workspaces and fast dynamic responses.

Pokines et al. developed a micro-electro-mechanical system (MEMS) based on bridge-type micro amplification with a DAR of 5.48 [12], which can provide larger DAR with more compact mechanism dimension. Sohail et al. proposed an analysis method of MEMS-based displacement amplification mechanism and proved the relationship between structural parameters and DAR [13]. Xu et al. investigated a flexure-based compound bridge-type displacement amplifier for PZT drives; the DAR was 12.80, the initial natural frequency was 89.19 Hz, and the output displacement was over 1 mm [14].

Most high-precision positioning platforms have a small range of motion (<200 μm) [2,3,4,15]. The primary design motivation is to develop a compact nanopositioning platform with a motion range exceeding 300 μm and a maximized resonance frequency for atomic force microscopy applications [16].

A compact multi-displacement amplifier structure is designed by integrating a rhombus amplifier with a lever mechanism, thereby achieving a larger DAR. This configuration amplifies the PZT actuator’s output displacement, consequently extending the nanopositioning platform’s motion range. Moreover, a void-cut strategy is employed to eliminate unnecessary mass, which significantly elevates the natural frequency of the nanopositioning stage. Additionally, decoupling beams are incorporated to mitigate output crosstalk [15].

Piezoelectric materials exhibit significant nonlinear rate-dependent hysteresis behaviors that considerably degrade their positioning accuracy, especially under high-frequency excitations [17,18]. Rate-dependent hysteresis models, particularly differential-based approaches such as the Bouc–Wen and Dahl models, have been developed to characterize these behaviors. Ao et al. developed a dual-structure reinforced MXene/PVDF-TrFE piezoelectric nanocomposite that achieves significant enhancement in piezoelectric properties through the synergistic coupling of interfacial polarization and structural engineering. The remarkable improvement in piezoelectric current output—representing a threefold boost in piezoelectric response—offers a promising strategy for addressing the rate-dependent nonlinear hysteresis in piezoelectric stack actuators [19].

The remainder of this paper is organized as follows. Section 2 introduces the structure design and a pseudo-static model is applied to analyze the flexible mechanism. Section 3 uses the ANSYS Workbench 19.2 to verify the performance of the designed model. The proposed prototype is tested in Section 4, and finally, the conclusion is provided in Section 5.

## 2. Design and Kinematics Modeling

**Figure 1:**
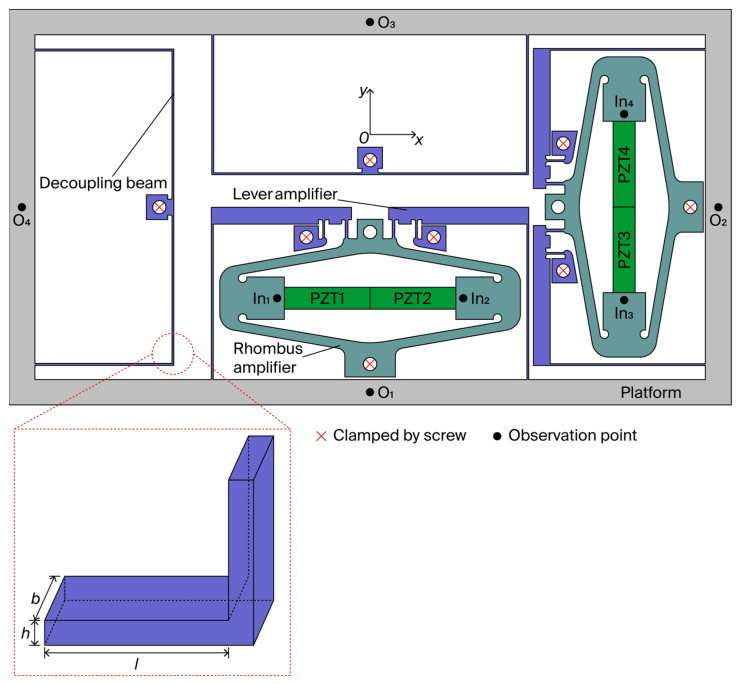
Proposed 2-DOF nanopositioning stage.

The design of the rhombus and lever amplifiers is shown in Figure 1. The rhombus amplifier offers the dual benefits of preloading the PZT actuator and providing displacement amplification, while its integration with a lever amplifier yields a compact architecture that further extends the stroke. Decoupling beams are incorporated to fully suppress crosstalk between the output displacements along the x- and y-axes.

A pseudo-static model is adopted to describe concurrent kinematics and dynamics of the entire flexible mechanism [20]. Assuming linear elastic deformation, as illustrated in Figure 1, the mechanism is mainly composed of flexible beams. Evidently, different structures of flexible beams have different stiffness and bear different degrees of deformation. The compliance of the flexible beam is considered to improve the modeling accuracy.

**Figure 2:**
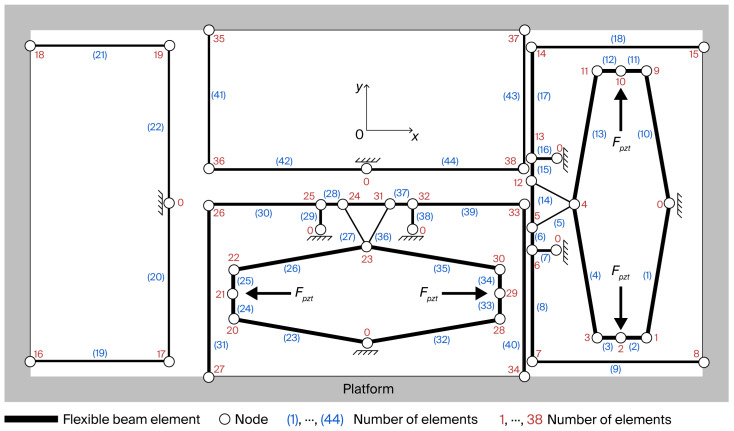
Discretization and numbering of the entire mechanism.

As illustrated in Figure 2, the proposed 2-DOF nanopositioning stage is simplified and represented as a series of flexible beams, indexed from (1) to (44). Nodes numbered 1 to 38 serve as connectors between adjacent beams, while all fixed nodes are designated as node 0. The PZT actuator’s output force is denoted as *F_pzt_*. The input beam, which interfaces directly with the PZT actuator, is modeled as a flexible beam due to its compliance exerting a significant influence on the static and dynamic characteristics of the entire mechanism.

**Figure 3:**
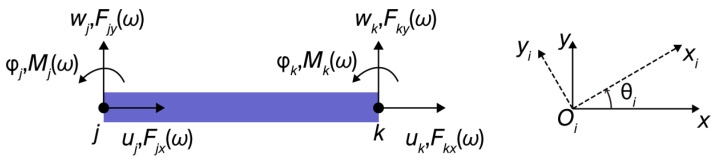
Nodal displacement and nodal force of the flexible beam.

The flexible beam is idealized as simplified rod element with uniform cross-sectional geometry in Figure 3. In this discretized representation, each rod element is defined by a pair of terminal nodes, denoted as *j* and *k*. Within the local coordinate system, each node possesses three independent DOFs: axial displacements ($u_{j}$ and $u_{k}$), transverse deflections ($w_{j}$ and $w_{k}$), and rotations about the out-of-plane axis ($\varphi_{j}$ and $\varphi_{k}$), corresponding to the forces at the node.

Prior to establishing the pseudo-static model of the entire mechanism, the dynamic stiffness matrix of the flexible beam, which characterizes the frequency-dependent nodal force–displacement relationship, is derived. The dynamic stiffness matrix $D^{b}(\omega )$ of the flexible beam under the excitation frequency ω is expressed in Equation (1).

(1) $$D^{b}(\omega )=\left[\begin{array}{cccccc} d_{1} & 0 & 0 & d_{5} & 0 & 0\\ 0 & d_{2} & -d_{3} & 0 & d_{6} & d_{7}\\ & & d_{4} & 0 & -d_{7} & d_{8}\\ & & & d_{1} & 0 & 0\\ & sym & & & d_{2} & d_{3}\\ & & & & & d_{4} \end{array}\right],$$

In which

(2) $$\left\{\begin{array}{c} d_{1}=EA\alpha \cot\alpha /l,d_{2}=EI\beta^{3}(\cos\beta \sinh\beta +\sin\beta \cosh\beta )/Rl^{3},d_{3}=EI\beta^{2}(\sin\beta \sinh\beta )/Rl^{2},\\ d_{4}=EI\beta (\sin\beta \cosh\beta -\cos\beta \sinh\beta )/Rl,d_{5}=-EA\alpha \cot\alpha /l,d_{6}=EI\beta^{3}(\sin\beta +\sinh\beta )/Rl^{3},\\ d_{7}=EI\beta^{2}(\cosh\beta -\cos\beta )/Rl^{2},d_{8}=EI\beta (\sinh\beta -\sin\beta )/Rl,R=1-\cos\beta \cosh\beta \end{array}\right.$$

where $\alpha^{2}=\omega^{2}l^{2}\rho /E$ and $\;\beta^{4}=\omega^{2}l^{4}\rho A/EI$. The parameter E denotes the Young’s modulus, while ρ represents the mass per unit length, and l specifies the length of the flexible beam. The cross-sectional area A and the second moment of area about the neutral axis I are given by *bh* and *bh*^3^/12, respectively, where *b* and *h* correspond to the width and height of the beam’s cross-section, as illustrated in Figure 1.

Utilizing the dynamic stiffness matrix of the flexible beam expressed in Equation (1), the nodal force–displacement relationship of the flexible beam within the reference system can be articulated as follows:

(3) $$\left\{\left.\begin{array}{c} F_{i,j}(\omega )\\ F_{i,k}(\omega ) \end{array}\right\}\right.=D_{i}(\omega )\cdot \left\{\left.\begin{array}{c} x_{j}\\ x_{k} \end{array}\right\}\right.=\left[\begin{array}{cc} k_{i,1} & k_{i,2}\\ k_{i,3} & k_{i,4} \end{array}\right]\cdot \left\{\left.\begin{array}{c} x_{j}\\ x_{k} \end{array}\right\}\right.,$$

Here, $F_{i,j}\left(\omega \right)={\left[\begin{array}{ccc} F_{jx}, & F_{jy}, & M_{j} \end{array}\right]}^{T}$ and $F_{i,k}\left(\omega \right)={\left[\begin{array}{ccc} F_{kx}, & F_{ky}, & M_{k} \end{array}\right]}^{T}$ denote the two nodal forces, while $x_{j}={\left[\begin{array}{ccc} u_{j}, & w_{j}, & \varphi_{j} \end{array}\right]}^{T}$ and $x_{k}={\left[\begin{array}{ccc} u_{k}, & w_{k}, & \varphi_{k} \end{array}\right]}^{T}$ represent the two nodal displacements, which are all defined within the reference coordinate system. [$k_{i,1}$, $k_{i,2}$;$\;k_{i,3}$,$\;k_{i,4}$] is the dynamic stiffness matrix for the *ith* flexible beam, transformed from the local coordinate system via rotational transformation.

(4) $$Di(\omega )=R_{i}^{T}\cdot D^{b}(\omega )\cdot R_{i},$$

where the rotation matrix $R_{i}$ is defined based on the orientation of the *ith* flexible beam relative to the reference coordinate system, and $\theta_{i}$ is the angle from the global *x*-axis to the local *x_i_*-axis, as illustrated in Figure 3.

(5) $$R_{i}=\left[\begin{array}{cccccc} \cos\theta_{i} & \sin\theta_{i} & & & & \\ -\sin\theta_{i} & \cos\theta_{i} & & & & \\ & & 1 & & & \\ & & & \cos\theta_{i} & \sin\theta_{i} & \\ & & & -\sin\theta_{i} & \cos\theta_{i} & \\ & & & & & 1 \end{array}\right],$$

Considering each node (from 1 to 38) as the research object, the force applied on the node is the sum of the inverse nodal forces from its connected flexible beams and any external force. Consequently, for each node, the following force equilibrium equation can be derived for the *nth* node:

(6) $$\sum_{N}\left[F_{i,j}(\omega )\;\mathrm{or}\;F_{i,k}(\omega )\right]=P_{n},$$

where *N* is the total count of flexible beams interfacing with the *nth* node. As illustrated in Figure 2, the *nth* node may be linked to the *j*-end or *k*-end of the *ith* flexible beam. Consequently, when the *nth* node is linked to the *j*-end, then $F_{i,j}\left(\omega \right)$ is applicable; otherwise, $F_{i,k}\left(\omega \right)$ becomes relevant. *P_n_* represents the aggregate of external forces directly exerted on the *nth* node. For example, the output force of PZT actuator *F_pzt_* is presumed to be directly applied to nodes 2, 10, 21, and 29.

Equation (3) is used to transform Equation (6) into the following:

(7) $$\sum_{N}\left[(k_{i,1}x_{j}+k_{i,2}x_{k})\;\mathrm{or}\;(k_{i,3}x_{j}+k_{i,4}x_{k})\right]=P_{n},$$

Equation (7) represents the force equilibrium equation at the *nth* node. Taking node 21 in Figure 2 as an example,

(8) $$k_{24,3}x_{20}+x_{24,4}x_{21}+k_{25,1}x_{21}+k_{25,2}x_{22}={\left[\begin{array}{ccc} -F_{pzt} & 0 & 0 \end{array}\right]}^{T}.$$

Equilibrium equations for the remaining nodes can be derived analogously from Equation (7), and are therefore omitted for brevity. By referring to the equilibrium equations of all nodes in turn, the pseudo-static model of the flexible mechanism with node displacements as the primary variables can be constructed as follows:

(9) D(ω)·X=F(ω),

where $X=\left[x_{20};x_{21};x_{22};\cdots ;x_{38}\right]$ and $F(\omega )=\left[P_{20};P_{21};P_{22};\cdots ;P_{38}\right]$. The dynamic stiffness matrix D(ω) of half of the mechanism in the *y*-direction is as follows:

(10) $$D(\omega )=\left[\begin{array}{ccccc} k_{23,4}+k_{24,1} & k_{24,2} & 0 & \cdots & 0\\ k_{24,3} & k_{24,4}+k_{25,1} & k_{25,2} & \cdots & 0\\ \vdots & \vdots & \vdots & \vdots & \vdots \\ 0 & 0 & 0 & k_{43,3} & k_{43,3}+k_{43,4} \end{array}\right],$$

With a similar derivation procedure, the dynamic stiffness matrix in the *x*-direction can be obtained but is omitted here for the symmetry of the mechanism. By setting the dynamic frequency ω to 0, the DAR, *A*, in the *y*-direction can be calculated as follows:

(11) $$A=\frac{x_{27(y)}}{2x_{21(x)}},$$

where the variable $x_{27(y)}$ corresponds to the output displacement along the *y*-direction, while $x_{21(x)}$ corresponds to the input displacement, as illustrated in Figure 2.

## 3. Finite Element Analysis and Evaluation

The static performance and natural frequency were analyzed by ANSYS Workbench 19.2 to verify the feasibility of the designed model. The material of the mechanism was Al7075 [21] with Young’s modulus of 71.7 GPa, density of 2770 kg/m^3^, Poisson’s ratio of 0.33, and yield strength of 503 MPa.

### 3.1. Static Structural Analysis

**Figure 4:**
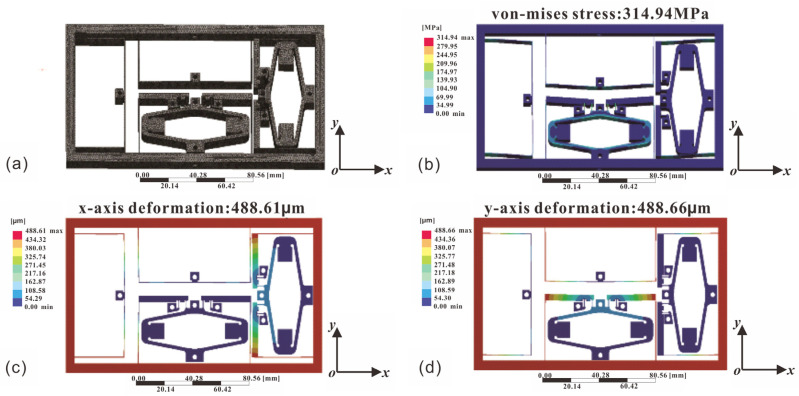
Finite Element Analysis (FEA) results for static structure analysis. (**a**) Three-dimensional FEA model, (**b**) von Mises stress distribution, (**c**) deformation along the x-axis, (**d**) deformation along the y-axis.

The three-dimensional FEA model was established as illustrated in Figure 4a.

(1) To assess the amplification capability and decoupling performance of the modeled stage under an x-axis driving force, the input forces are applied at points *In*_1_ and *In*_2_. The subsequent deformation characteristics are quantified as follows: the ratio of *do*_1_*y*, *do*_2_*y*, *do*_3_*y*, and *do*_4_*y* captures the deformation relations at the respective points, while the sum of *dIn*_1_*x* and *dIn*_2_*x* denotes the relationship between the stage’s *y*-axis output displacement and the actuator’s output displacement. The parasitic movements at the output points are denoted by *do*_1_*x*, *do*_2_*x*, *do*_3_*x*, and *do*_4_*x*, and the *y*-axis output coupling error is defined as $Error1\;=\;\sum do_{i}x/\sum do_{i}y(i=1,2,3,4)$.

(2) In the same way, the input forces on points *In*_3_ and *In*_4_ are adopted to assess the effect of a y-axis driving force. The ratio of *do*_1_*x*, *do*_2_*x*, *do*_3_*x*, and *do*_4_*x* captures the deformation relations at the respective points along the x-axis, while the sum of *dIn*_3_*y* and *dIn*_4_*y* denotes the relationship between the stage’s *x*-axis output displacement and the actuator’s output displacement. The parasitic movements at the output points are denoted by *do*_1_*y*, *do*_2_*y*, *do*_3_*y*, and *do*_4_*y*, and the *x*-axis output coupling error is defined as $Error2\;=\;\sum do_{i}y/\sum do_{i}x(i=1,2,3,4)$.

Subsequently, a series of forces were applied to points *In*_1_ and *In*_2_ along the x-axis (Table 1) to assess the stage’s accuracy, followed by the application of an identical force set to points *In*_3_ and *In*_4_ along the y-axis, as detailed in Table 2. The results demonstrate that, with increasing actuation force, the displacements of all critical points exhibited a proportional increase, thereby confirming the linear relationship between the input force and the stage’s output displacement. When a driving force was applied to points *In*_1_ and *In*_2_ along the x-axis, the maximum deformation at the output of the stage occurred at point *O*_1_, with an average output coupling error of 0.336%. The *y*-axis displacement amplification ratio Ay of the stage was 14.18. Conversely, applying the force to points *In*_3_ and *In*_4_ along the *y*-axis resulted in the maximum deformation at point *O*_2_, with an average output coupling error of 0.24%. The observed discrepancy may be attributed to elastic deformation. The x-axis displacement amplification ratio Ax of the stage was determined to be 14.36.

Exerting 1600 N force on points *In*_1_ and *In*_2_ by simulating the maximum output force of piezoelectric actuator, as depicted in Figure 4b, indicates a peak von Mises stress of 314.94 MPa (well below the material’s yield strength of 503 MPa). The corresponding maximum deformations in the x- and y-axis are 488.61 µm and 488.66 µm, presented in Figure 4c,d, respectively.

### 3.2. Modal Analysis

**Figure 5:**
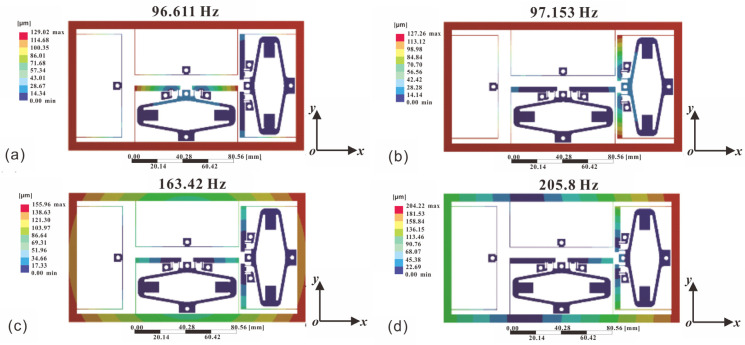
Results of modal analysis. (**a**) Mode 1: 96.611 Hz, (**b**) Mode 2: 97.153 Hz, (**c**) Mode 3: 163.42 Hz, (**d**) Mode 4: 205.8 Hz.

As illustrated in Figure 5, the deformation patterns associated with Modes 1 and 2 manifest along the y- and x-axes, respectively. Owing to the near-symmetrical nature of the structure, the deformation responses in the x- and y-directions are closely matched, resulting in adjacent resonance frequencies of 96.611 Hz and 97.153 Hz. Conversely, Modes 3 and 4 correspond to rotational and shear deformation, with resonance frequencies of 163.42 Hz and 205.8 Hz. Evidently, the x–y nanopositioning stage primarily operates under applied forces that induce deformation within the x–y plane. Consequently, the initial two natural frequencies of the stage are pivotal to the overall positioning performance.

## 4. Experimental Implementation

**Figure 6:**
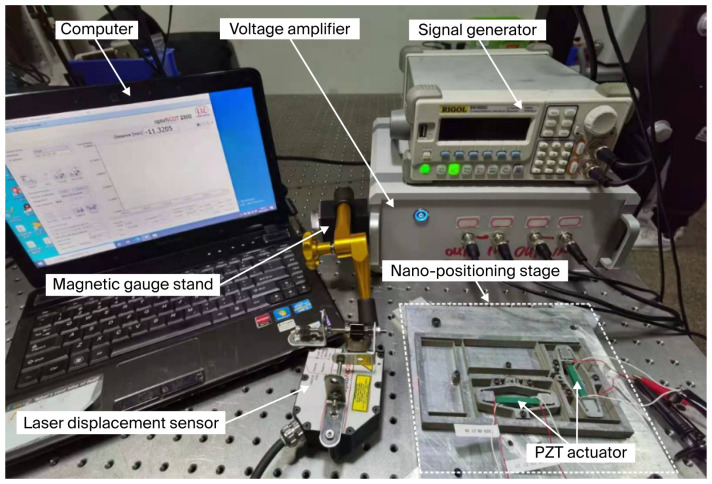
Experimental setup of the proposed nanopositioning stage.

A specimen of AL7075-T651 alloy (Sichuan Jiuda New Material Technology Co., Ltd., Guangyuan, Sichuan, China) was selected for the fabrication of the experimental prototype utilizing the wire electrical discharge machining (WEDM) process. The comprehensive experimental setup is illustrated in Figure 6. The nanopositioning stage was driven by four PZT actuators (Pst150/5×5/20, Harbin, China) with d33 = +635 pm/V and maximum output force 1600 N, which were powered by a dual-channel voltage amplifier. Precise displacement measurements of the stage were acquired using a laser displacement sensor (LTS-025-04, MTI Instruments, Albany, NY, USA). To mitigate the influence of external disturbances, all equipment was mounted on a vibration isolation platform.

**Figure 7:**
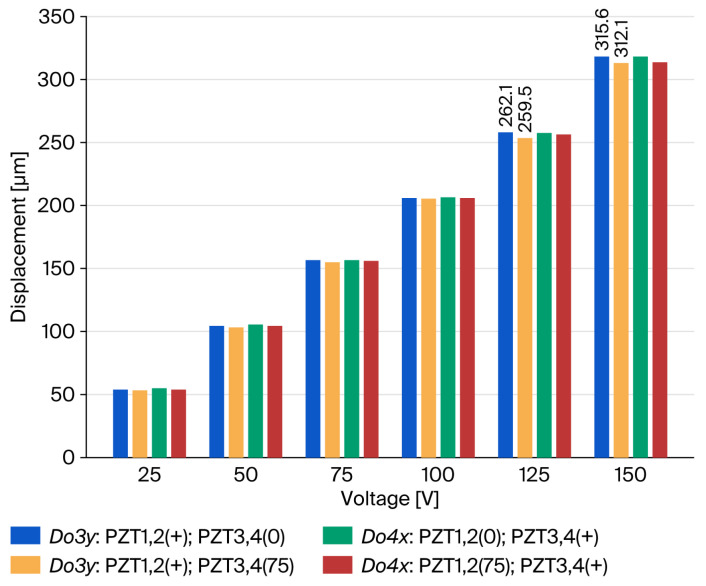
Crosstalk test result.

Several open-loop experiments were conducted. The preload on the PZT actuator was 500 N; a voltage sweep ranging from 0 to 150 V was sequentially applied to PZT1, PZT2, PZT3, and PZT4. The resultant displacement responses at points *O*_3_ and *O*_4_ were recorded at a constant sampling interval. Four distinct sets of experimental data were obtained and are presented in Figure 7. *Do3y* denotes the output displacement detected at *O*_3_ in the *y*-direction, and *Do4x* denotes the output displacement detected at *O*_4_ in the *x*-direction. (+) denotes the PZT actuated with changed voltage (0–150V), (0) denotes that PZT was not actuated, and (75) denotes the PZT actuated with a constant voltage (75V). As illustrated in Figure 7, the *x*/*y*-axis drive input displacement slightly influences the *y*/*x*-axis drive output displacement.

**Figure 8:**
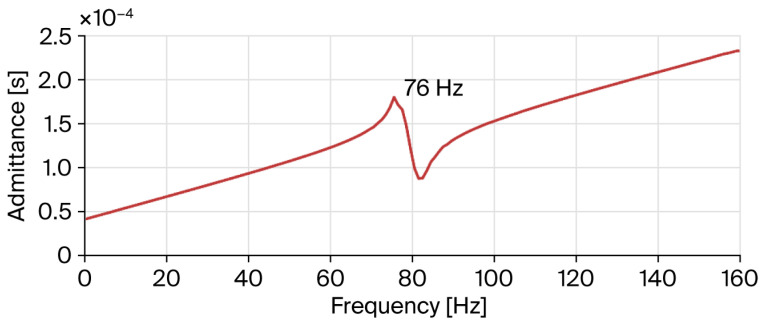
Admittance curve of the nanopositioning stage.

**Figure 9:**
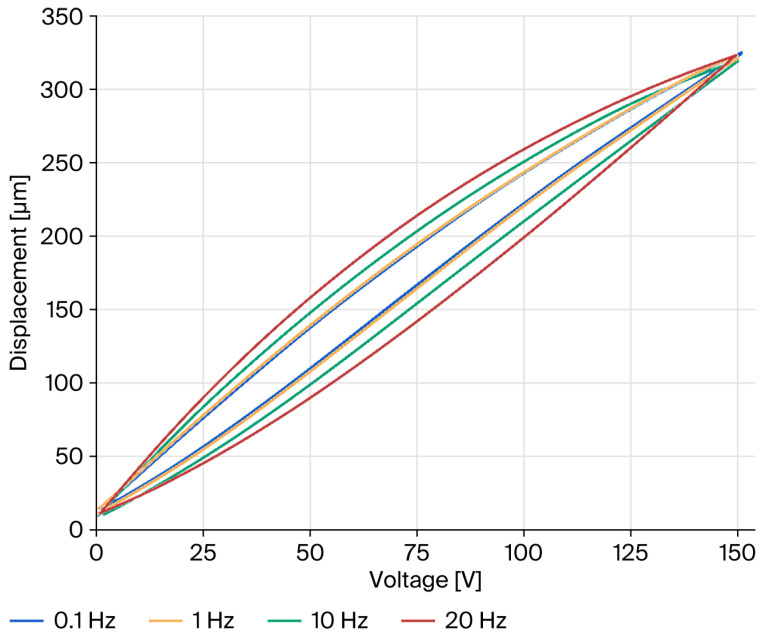
Open-loop hysteresis characteristics across different voltage frequencies.

The dynamic performance of the nanopositioning stage was characterized using an impedance analyzer to corroborate its operational efficacy. As illustrated in Figure 8, a notable inflection point emerges at approximately 76 Hz, indicating that the actual resonance frequency is very close to the simulated prediction of 78.4 Hz. In general, the discrepancy between the theoretical material properties of the prototype model and the actual manufacturing tolerances, as well as defects introduced during fabrication, can result in significant deviations between the actual resonance frequency and the predicted value. Empirical data correlating output displacement with applied voltage were systematically recorded and illustrated in Figure 9. Experimentally, under a preloading force of 500 N and a maximum drive voltage of 150 V, compared with an estimated displacement output about 336 μm × 336 μm, the PZT actuator archived a workspace of 312 μm × 312 μm, which aligned with the simulated displacement output.

The testing protocol involved the application of a series of non-negative triangular voltage waveforms at frequencies of 0.1 Hz, Hz, 10 Hz, and 20 Hz to the PZT actuators. The results elucidate a pronounced rate dependency of the hysteresis phenomenon: higher excitation frequencies engender broader hysteresis loops, indicative of exacerbated nonlinearity. While the mitigation of hysteresis nonlinearity lies beyond the scope of this investigation, it is amenable to amelioration through the implementation of a closed-loop feedback control strategy.

**Figure 10:**
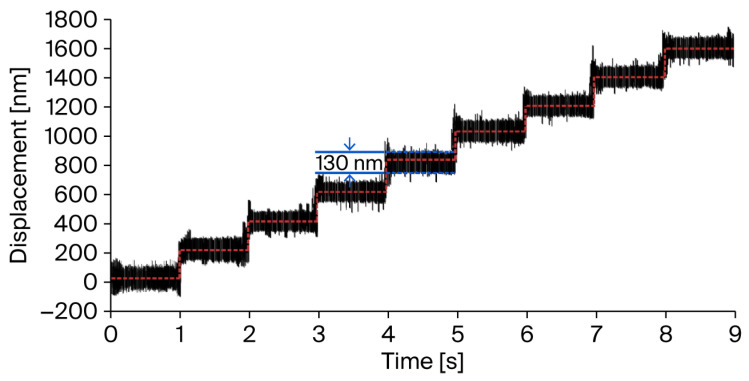
Minimum resolution test result.

As illustrated in Figure 10, in typical open-environment conditions, the multistep response with a 130 nm step size was effectively resolved with open-loop feedback control. In this case, the minimum resolution was constrained by the sensor’s peak-to-peak noise of 130 nm@40 kHz.

When evaluating the performance of a PZT nanopositioning platform, it is essential to consider a range of performance metrics, including stroke, resonant frequency, resolution, coupling error, and dimension of the mechanism. These performance parameters are often interdependent, rendering direct comparisons between individual stages challenging. To facilitate an objective assessment, the concept of “area efficiency” (defined as the ratio of workspace area to mechanism area) introduced in reference [22] is employed. A higher area efficiency indicates a more compact and thus more effective nanopositioning stage. For a comprehensive comparison with the proposed PZT nanopositioning stages, several representative mechanisms are summarized in Table 3. The area efficiency value of the proposed stage is much higher than the other stages, confirming its superior performance and suitability for miniaturization.

## 5. Conclusions

The detailed design of an XY PZT nanopositioning platform is presented. A compact multi-stage amplification mechanism, integrating a rhombus amplifier and a lever amplifier, is introduced to augment the platform’s workspace. A pseudo-static model was employed for dynamic modeling analysis of the entire system, transforming the dynamic modeling of the compliant mechanism into a static-like problem, thereby simplifying the modeling process for complex compliant mechanisms. The performance of the established model was validated using FEA software (ANSYS Workbench 19.2). The prototype machine was independently manufactured and tested. Experimental results demonstrate that the stage achieves a workspace of 312 μm × 312 μm, a fundamental frequency of 76 Hz, and compact dimensions of 173.5 mm × 93 mm × 10 mm. The experimental results show that the PZT platform can be widely used in the field of precise positioning, and demonstrate the platform’s potential for further miniaturization. As precise rate-dependent hysteresis models are promising to solve frequency-dependent effects of PZT actuators, future research will focus on the implementation of hysteresis compensation and frequency response analysis, which is essential for high-precision positioning applications.

## Figures and Tables

**Table 1:** Static structure analysis of data of x-axis output.

Parameters	100 N	200 N	300 N	400 N	500 N
*Din*_1_*x* (μm)	3.8265	7.653	11.479	15.306	19.132
*Din*_2_*x* (μm)	3.8264	7.6528	11.479	15.306	19.132
*Do*_1_*y* (μm)	110.66	221.31	331.97	442.62	553.28
*Do*_1_*x* (μm)	0.046787	0.093574	0.14036	0.18715	0.23393
*Do*_2_*y* (μm)	108.24	216.48	324.72	432.96	541.2
*Do*_2_*x* (μm)	0.49371	0.98742	1.4811	1.7848	2.4686
*Do*_3_*y* (μm)	107.11	214.21	321.32	428.42	535.53
*Do*_3_*x* (μm)	0.23527	0.47053	0.7058	0.94107	1.1763
*Do*_4_*y* (μm)	108.14	216.29	324.43	432.57	540.71
*Do*_4_*x* (μm)	0.68333	1.3667	2.05	2.7333	3.4167
*Ay*	14.1823	14.1823	14.1828	14.1821	14.1825
*Error*1	0.336%	0.336%	0.336%	0.336%	0.336%

**Table 2:** Static structure analysis of data of y-axis output.

Parameters	100 N	200 N	300 N	400 N	500 N
*Din*_3_*y* (μm)	3.8239	7.6477	11.472	15.295	19.119
*Din*_4_*y* (μm)	3.8176	7.6353	11.453	15.271	19.088
*Do*_1_*y* (μm)	0.10949	0.21897	0.32846	0.43795	0.54743
*Do*_1_*x* (μm)	109.73	219.45	329.18	438.9	548.63
*Do*_2_*y* (μm)	0.19867	0.39734	0.59602	0.79469	0.99336
*Do*_2_*x* (μm)	109.97	219.93	329.9	439.86	549.83
*Do*_3_*y* (μm)	0.32981	0.65961	0.98942	1.3192	1.649
*Do*_3_*x* (μm)	109.59	219.18	328.76	438.35	547.94
*Do*_4_*y* (μm)	0.41579	0.83158	1.2474	1.6632	2.079
*Do*_4_*x* (μm)	109.69	219.39	329.08	438.77	548.46
*Ax*	14.3604	14.3615	14.3612	14.3532	14.3616
*Error*2	0.24%	0.24%	0.24%	0.24%	0.24%

**Table 3:** Performance comparison of the nanopositioning stage.

Ref.	Dimension (mm^2^)	DAR	Bandwidth (Hz)	Workspace (μm^2^)	Area Efficiency
[15]	220 × 220	1	719.49	19.2 × 18.8	0.01
[16]	167.9 × 167.9	4.2	831	119 × 121.4	0.51
[21]	98 × 56	12.84	83	963 μm	0.18
[23]	92 × 50	12.1	205	214 μm	0.05
This Paper	173.5 × 93	9.3	76	312 × 312	6.03

## Data Availability

The data that support the findings of this study are available on request from the corresponding author, upon reasonable request.
